# Effects of the utilization of intellectual property by scientific researchers on economic growth in Mexico

**DOI:** 10.1371/journal.pone.0258131

**Published:** 2021-10-13

**Authors:** Luis Felipe Beltrán-Morales, Marco Antonio Almendarez-Hernández, Gerzaín Avilés-Polanco, David J. Jefferson

**Affiliations:** 1 Centro de Investigaciones Biológicas del Noroeste (CIBNOR), La Paz, Mexico; 2 CONACYT-Centro de Investigaciones Biológicas del Noroeste (CIBNOR), La Paz, Mexico; 3 School of Law, the University of Canterbury, Christchurch, Aotearoa, New Zealand; Universidad Veracruzana, MEXICO

## Abstract

The present article examines the impact of intellectual property (IP) utilization and concentration on economic growth in Mexico. The findings presented center on the use of different forms of IP by researchers in the National System of Researchers (SNI in Spanish) of Mexico. We focus especially on the externalities associated with the use of IP by researchers, as well as on understanding how knowledge about, and utilization of IP relates to economic growth, as measured by gross domestic product (GDP). The results of our analyses indicate that in the context of the Mexican SNI, the utilization of certain forms of IP, specifically patents and industrial designs, had a positive impact on economic growth, while the use of utility models was negatively linked to drivers of growth. Policies based on these results could seek to foster awareness and utilization of particular forms of IP by SNI researchers, which in turn could result in greater economic growth in Mexico.

## Introduction

Many recent studies have focused on the role that intellectual property (IP) plays in economic growth in different countries. For instance, Boldrin and Levine [1] trace the influence of IP on wealth generation to factors including globalization, which has facilitated the development of more complex and specialized products and services more quickly. Other dynamics that have been identified as relevant include the increased mechanization of production processes, as well as the importance of creative and innovative activity in generating value-added products.

These factors have contributed to economic growth in many countries, frequently intertwining with increasing rates of IP utilization beginning especially in the 1980s. Some scholars have traced the expansion of applications for IP to changes in law and practice in territories such as the United States or Europe [2]. Regardless of the origins of this trend, IP has become increasingly relevant worldwide, for instance as the result of activities spearheaded by entities such as the World Intellectual Property Organization (WIPO), and obligations under multilateral treaties such as the Agreement on Trade-Related Aspects of Intellectual Property (TRIPS) of the World Trade Organization [3].

Prior empirical studies have demonstrated that when effective, legal frameworks granting IP rights can positively impact long-term economic growth rates. Such findings have been attributed to the notion that enforceable monopolistic rights may encourage greater investments in scientific research, technological development, and innovation [4–9]. Meanwhile, economists have advocated for employing data associated with rates of IP protection as a metric for the evaluation of collaborative research projects [10].

The rationale behind such arguments is that in comparison to research products that are protected as IP, greater uncertainty exists in relation to how to effectively manage and use public goods. Where there is a lack of clarity surrounding the rights and obligations of the diverse parties collaborating in research projects, scientific partnerships may be negatively impacted, which could also detrimentally affect the potential downstream economic impacts that research and development projects may generate.

In recent years, various forms of IP (e.g., patents, plant breeders’ rights) have been increasingly regarded as one of the essential outputs of successful scientific research projects. This is because IP functions as a catalyst for transferring research products from theoretical knowledge to commercial applications [11–15]. Patents in particular are often used as proxies to measure inventive or innovative activity [16, 17]. Likewise, patent applications can be used as a metric for evaluating innovation in situations where long gaps in time exist between the filing of patent applications and the granting of rights, which may be the case in many developing countries [18].

Both neoclassical [19, 20] and modern economic theories [21–23] generally regard technical progress as one of the principal determinants in economic growth. In this context, economists have defined innovation broadly, to include technological advances, applied research, and improvements in social welfare and administrative processes, among others. It is therefore appropriate that different forms of IP protection exist for diverse embodiments of innovative products and processes.

Today, the most common forms of IP include copyright, patents, industrial designs, utility models, trademarks, and trade secrets [24]. The present analysis focuses specifically on the utilization and concentration of IP as granted through patents, industrial designs, and utility models. Generally, patents protect new and non-obvious (or non-incremental) inventions, which are defined as products and processes derived from human ingenuity, such that once put into practice the protected subject matter will manifest in a tangible form. Meanwhile, industrial designs protect ornamental or aesthetic aspects of an object, which can include three dimensional features such as shape or two-dimensional features such as patterns, lines, or colors. Finally, utility models offer a form of IP for minor (or incremental) improvements of previously existing products. Utility models, also known as “petty patents,” are not offered in all countries, but historically they have been widely used in Mexico.

The National Council of Science and Technology (CONACYT, for its acronym in Spanish) is the institution responsible for promoting the development of science, technology and innovation throughout Mexico. The National System of Researchers (SNI), which is part of CONACYT, is an organization that provides various incentives to its members, including in relation to the filing of patent applications. The rationale for this is that in Mexico, patent filings are conceptualized as an indicator that forms part of the criteria used to evaluate entrance into the SNI, in addition to renewal of membership and promotion within the System. In return for obtaining patents, Mexican researchers receive a monthly payment from CONACYT.

Other kinds of research outputs based on which SNI affiliates are evaluated include the publication of scientific articles, book chapters, and books. The number of publication citations and engagement in other professional academic activities (teaching undergraduate and postgraduate classes, Master’s and doctorate thesis supervision) are also relevant criteria for the evaluation of scientists. Mexican researchers primarily obtain funding to undertake scientific and technological activities through various calls for proposals emitted by CONACYT and other public institutions. During the period studied for the present project, in order to qualify for membership in and obtain monthly payments from the SNI, Mexican scientists needed to be employed in a public or private institution in which they worked at least 40 hours per week on activities in scientific or technological fields. Since that time, the policy was changed such that today, only scientists employed in Mexican public educational or research institutions are eligible to receive the monthly payment, although researchers working in private universities or research institutes are still eligible to receive non-economic distinctions from the SNI.

More than 50% of the national R&D expenditure in Mexico is publicly funded [25], and of patents granted to research institutions, 95% are filed by public institutions [26]. As in other countries, one way to measure inventive activity in Mexico is to examine the volume of IP filings, including for patents, utility models, and industrial designs. Notably, however, the number of applications lodged under these three IP frameworks is lower in Mexico in relation to higher income countries. This may be explained by the fact that in general, the objectives, undertaking, and results of scientific research projects conducted in Mexican public institutions do not respond to market needs, but rather are designed to generate social and cultural well-being [27, 28].

According to Amigo [29], one reason that the patenting activity of SNI members is low is that the process of patent examination by the Mexican Institute of Industrial Property (IMPI) takes approximately four years, while the review and publication of a scientific journal article typically occurs within one year. Similar to patents, the examination of applications for utility models and industrial designs may require up to four years [28]. Given the relatively long examination times for the prosecution of IP applications and the fact that evaluation within the SNI generally occurs every three to five years depending on a given scientist’s level of appointment, Mexican researchers may prefer to pursue scientific publications rather than patents, utility models, or industrial designs as research outputs. During the period in which the present study was conducted, industrial designs were the most common form of IP sought by Mexican inventors (65–75%), followed by patents (15–22%), and finally utility models (8–15%) [30].

Despite the challenges that researchers in Mexico face when seeking IP in relation to their work, they are nevertheless encouraged to lodge applications for patents, utility models, and industrial designs. For instance, IMPI has created numerous tools and services that are designed to enhance the ability of researchers and administrators from the SNI to participate in the national IP system. These include technological information searches such as national and international bibliographic reviews; national and international technical information searches; state of the art searches; selective information reviews; and monitoring related to specific technological areas. Of the total number of technological information searches conducted by IMPI from 2003 to 2012, between 98 and 100% were done for inventors working at Mexican institutions [30]. These searches are intended to support decisions related to the commercialization of research results, for instance surrounding whether a particular invention has sufficient market potential, or about the timing of when a product should be launched. Such information can also enable inventors and technology managers to better understand the relevant state of the art, thereby improving their chances of obtaining IP rights following examination by IMPI.

In the context of SNI researchers’ interactions with IMPI, for the present study we proposed the following hypotheses: 1) The Mexican states with the largest numbers of SNI members tend to generate greater inventive activity, which in turn leads to achieving higher rates of economic growth relative to other states; 2) The states with greater relative importance in the structure of their IP measured through concentration indexes tend to achieve higher rates of economic growth relative to other states; 3) The states where the Mexican inventors responsible for the greatest proportion of technological information searches are located demonstrate higher rates of economic growth relative to other states; 4) The states with the greatest capacity to accept the transmission and diffusion of positive externalities achieve higher rates of growth relative to states that are less adept at these activities.

## Theoretical basis for the present study

The traditional neoclassical economic theory and the theory of endogenous growth have both postulated that technological change is one of the principal determinants of economic growth. The field of neoclassical economics has focused since its inception on uncovering the roots of innovation, based on the pioneering work of Abramovitz [19] and Solow [20]. This latter economist found that the rate of long-term growth was primarily influenced by the “Solow residual,” which he conceptualized as technical change. According to this view, technology is not the result of decisions made by economic agents, but rather derives from invisible external factors that the model is not capable of explaining directly. As such, the primary limitation of Solow’s model is that in order to be measured, technical change must be introduced exogenously into econometrics models.

As a result of criticisms Solow’s model, the theory of endogenous growth subsequently gained traction through the works of economists such as Romer [31], Lucas [32], Barro [33], and Rebelo [34], whose research sought to identify a more sophisticated model to more precisely explain the long-term growth of economies through the use of endogenous variables. Particular attention was paid to human capital, the accumulation of knowledge, and public spending as factors that influence the forms that technical change might assume. Building on this work, second-generation models of the endogenous growth theory such as those developed by Romer [21, 35], Grossman and Helpman [22], and Aghion and Howitt [23], increasingly recognized the role that research and technological development play in a market structure of imperfect competition (monopoly). Therefore, under these models the main determinant of economic growth is understood to be technological change.

Accordingly, contemporary models based on endogenous growth theory conceive technological change as a causal factor driving the generation of new designs. These developments in turn result in improvements that enhance the competitiveness of productive processes, thereby fomenting continuous and dynamic growth. However, endogenous growth models are also limited in certain respects. According to Dutt [36], the most important drawback of these models is their limited capacity to consider the particular characteristics that are inherent to a given technology, as well as the institutional and cultural factors that affect technological change.

Empirical analyses using data from numerous world regions have found that variables such as research and development (R&D) expenditures, rates of technological innovation, and factors related to personnel working in the R&D sector are positively linked to economic growth. For instance, Bassanini and Scarpetta [37] used panel data focusing on pooled mean-group (MG) estimates from 21 member countries of the Organization for Economic Co-Operation and Development (OECD) for the years 1971–1998. The results found elasticities of 0.14 for total R&D expenditures, 0.13 for private R&D expenditures, and -0.37 for public R&D expenditures. The negative sign of this latter finding was attributed to the displacement of resources from the public to the private sector.

Similarly, Zachariadis [38] employed a system of three equations based on industrial manufacturing data from the United States, for the years of 1963–1988. Findings included elasticities ranging from 0.08–0.16, using the rate of product growth per worker as the dependent variable. Bayarcelik and Tasel [39] used a two-stage least squares (2SLS) regression analysis for the period of 1998–2010 in Turkey and obtained an elasticity of 0.015. However, some studies have offered contrasting findings. For instance, Birdhall and Rhee [40] found no statistical significance when assessing the impact of average R&D expenditures on GDP growth. The findings of this study were based on the results of an ordinary least squares (OLS) regression analysis for the period of 1970–1985, focusing on OECD member countries, as well as on developing countries. One reason that could explain the null findings in the Birdhall and Rhee [40] study is that the effect of R&D expenditures on economic growth could vary depending on whether a country is classified as “developed” or “developing.”

Notwithstanding the sometimes divergent findings associating R&D expenditures with GDP, one commonality across the majority of previous studies linking innovation and economic growth is that research has frequently focused on patents a proxy for measuring innovative activity that could drive economic growth [18, 41–49]. Very few analyses have focused on utility models as a form of IP that could be related to economic growth. This is likely due to the fact that utility models are only offered as a mechanism for IP protection in certain countries. While most research to date has found positive associations between patenting activity and economic growth, Bayarcelik and Tasel [39] found a negative effect of patenting on growth in Turkey. This result may be attributed to certain externalities that affect the process of IP protection in that country, such as high short-term costs associated with obtaining patents.

It is important to highlight that the scientific knowledge and technological developments generated in a particular region are not equally utilized by all economic actors. Nevertheless, the movement of innovations through the commercial flow of goods and services may result in positive externalities that affect a broader set of actors than those who directly benefit from local R&D efforts. For this reason, some studies have included as independent variables in their regression models factors such as R&D spending, total stock of technological capital, and patent applications, while examining growth in total productivity as the dependent variable of interest [50–54].

In the specific context of Mexico, prior economic analyses have examined the relationship between independent variables including investment in research, innovation (using patents as an indicator), and innovative capacity, with economic growth as the dependent variable [55–59]. Other studies, including those of Aboites and Díaz (2018) [60], Cepeda-Zetter *et al*. [61] and Meza-Rodríguez *et al*. [62], have examined patenting activity among Mexican inventors. Specifically, Aboites and Díaz [60] analyzed patenting behavior and observed patterns in the relationships between Mexican inventors and multinational companies. Among the principal results, it was found that following the entry into force of the North American Free Trade Agreement until 2016, there was an increase in the number of Mexican inventors who obtained patents granted by the United States Patent and Trademark Office and who licensed their commercial exploitation rights to non-Mexican entities.

Meanwhile, Cepeda-Zetter *et al*. [61] evaluated patent applications with a focus on gender using the PATENTSCOPE database. The results revealed that among Mexican researchers, male applicants tended to file patent applications as the sole inventor, whereas female applicants more commonly were named as part of a small or medium sized group of researchers in male dominated fields such as chemistry and metallurgy. For their part, Meza-Rodríguez *et al*. [62] focused on patenting activity at the local level in Mexico City. The results showed that in that jurisdiction nearly half of the patents granted were assigned to inventors who are Mexican residents, for inventions primarily classified as of a medium to high technological level.

Finally, other studies that examined IP activity in Mexico have used knowledge production functions from theoretical and empirical perspectives with specifications of count data, the standardized coefficient model and the estimator proposed by Driscoll and Craay. These analyses have generally found a positive relationship between SNI membership and patents [26, 28, 63, 64]. However, to date no studies have examined the relationship between IP as utilized by SNI researchers, externalities in the innovation ecosystem, and economic growth in Mexico. The present study was designed to address this gap in knowledge.

## Materials and methods

### Description of the econometric model and database utilized in the present study

Romer [21] argues that the impact of technological change on economic growth is related to investment decisions made by economic agents who seek to maximize benefits. The present study bases its assumptions on a similar theoretical and econometric model. We postulate that accumulated capital represents a mobile resource that may be transferred from the consumer sector to the capital goods sector for the purposes of producing new designs. The model is expressed through the Cobb-Douglas production function,

$$Y(H_{Y},L,x)=H_{Y}^{\alpha }L^{\beta }\sum_{i=1}^{\infty }x_{i}^{1-\alpha -\beta }$$ (1)

where *x* is an index of the innovative level of the technology, comprised of a combination of inputs directed towards the generation of a final product; *H* is human capital; and *L* is labor. Physical capital is defined as a set of intermediate goods and is measured in units consumed. It is further established in the production equation that all capital goods are not perfect substitutes. Instead, we assumed that capital goods have a separable additive function wherein capital goods are substitutes for other goods.

Given that technologies are introduced into the model as non-rival goods, the ideal market structure is not one in which firms are price-takers, but rather one where an environment of monopolistic competition is expected to occur. Therefore, an increase in market size: (1) incentivizes research; (2) increases income; (3) increases welfare; and (4) accelerates rates of economic growth.

In the theoretical model utilized in the present study, the knowledge that is crystallized in a new design is inserted into a given economy and affects production through two means: (1) it creates a good that is sacrificed for use in production; and (2) it enhances the total stock of knowledge and elevates the productivity of human capital in the R&D sector. Furthermore, our approach assumes that because the use of knowledge as an input is non-rivalrous, researchers take advantage of free access to the total stock of knowledge. Such access stimulates research wherein the technology is replicated, generating positive external effects through spillovers of knowledge. Therefore, although ownership of the property rights related to a given design used to produce a durable good is exclusive, the benefits that other economic agents derive from research activities related to a particular patent are not necessarily exclusive.

Given that durable goods are designated as a continuous variable, the Eq (1) is substituted by the following integral,

$$Y(H_{Y},L,x)=H_{Y}^{\alpha }L^{\beta }\int_{0}^{\infty }x{\left(i\right)}^{1-\alpha -\beta }di.$$ (2)

If in this integral the substitution x=K/nA, is made, the final production function is expressed as,

$$Y(H_{A},L,x)=(H_{Y}{A)}^{\alpha }(L{A)}^{\beta }({K)}^{1-\alpha -\beta_{n}\alpha +\beta -1}.$$ (3)

where n are the consumption units sacrificed (units of capital) to create a specified quantity of a durable good, and A is the stock of knowledge. In this way, technological level is associated with increasing returns to scale, caused by sustained increases in human capital and the total stock of knowledge.

To explore the impact that diverse forms of IP protections for innovations have on economic growth in Mexico, the present study was informed by the work of Grossman and Helpman [22] with respect to the equilibrium condition, where the present value of the inventor’s monopoly profits must equal the cost of the innovation. According to Solow (2000) the cost of invention can be described as follows,

$$\frac{aw}{k_{n}}$$ (4)

where *w* corresponds to salary, *a* is a parameter that represents the units of labor used in the innovation process and *k*_*n*_ represents the knowledge available, given the results of prior research and the contents of the public domain. Innovation increases *k*_*n*_ which makes research more productive, and therefore additional external effects are produced. It is assumed that there are *L* units of labor and that they are constant, that is, that there are no other sources of growth other than innovation. Following the exposition of Solow (2000), Thus, the equilibrium condition of the labor market would be,

$$\frac{a}{k_{n}}\hat{N}+X=L$$ (5)

where *a*/*k*_*n*_ is the quantity of labor that is required to make innovations and $\hat{N}$ is the number of current innovations, so $\frac{a}{k_{n}}\hat{N}$ corresponds to the quantity of labor that participates in research activities and *X* corresponds to the quantity of labor that is dedicated to the production of already known goods. This implies that economic growth is explained by an increase in research productivity, that is, *k*_*n*_ should grow over time. Research activity, in addition to innovations protected by monopoly IP rights, should create externalities that make research more productive, implying that *k*_*n*_ should be an increasing function of *N*. In this sense, Grossman y Helpman [22] assume that *k*_*n*_ is equal to *N*, so Eq (5) can be expressed as,

$$ak_{n}+X=L$$ (6)

The Fisher equation adjusted for research activity allows for the present value of profits generated from IP protection by innovation to be obtained, that is monopolized benefits at present value,

$$\Pi =(1-\alpha )\frac{pX}{N}$$ (7)

where *pX*/*N* represents the total income of the innovator, *α* is the fraction that corresponds to salary and (1−*α*) is the innovator’s profit. Meanwhile, the cost of innovation can be expressed as,

$$v=\frac{aw}{k_{n}}$$ (8)

Following the reasoning of Solow [65], “Stable growth requires that the present value of the innovator’s profits resulting from IP protection is equal to the cost of innovation adjusted to fit the fundamental trend and technological parameters of the model…” (p197), as well as, “technological parameters, and an economy that performs under the assumption of maximization of the inter-temporal utility of the representative consumer with a time preference rate *p* and an inter-temporal elasticity of substitution equal to a given constant…” ([65] (p197)).

Under this theoretical approach, the present study seeks to estimate the impact of the three forms of IP studied and membership in the SNI on the economic growth of Mexico. Given the costs of invention and the response time of the different protection modalities, innovators will seek to maximize their profits from their inventions under the form of IP protection that satisfies their inter-temporal preferences and thus impact on the economic growth of Mexico. To estimate these impacts, the following Cobb-Douglas type production function transformed into its log-linear form is proposed,

$${lngdp}_{it}=ln\beta_{0}+\sum_{j=1}^{N}B_{jit}lnX_{it}+u_{it}$$ (9)

where *lngdp* is the natural logarithm of the GDP, (based on 2008 data), *X*_*j*_ is a vector of input (1×*K*) labor, physical capital and human capital proxy variables including: members of the SNI per thousand members of the overall economically active population in Mexico; inventive activity for patents, utility models and industrial designs; technological indicators that measure externalities for patents, utility models and industrial designs; and concentration indexes of patents, industrial designs and utility models. *β* is a vector (1×*K*) of unknown parameters to be estimated. The description of the variables used to models 1–14 is shown in Table 1.

**Table 1:** Description of the variables.

Variable	Description	Source
lngdp	Natural logarithm of the GDP of Mexico, based on 2008 prices.	INEGI
lnfbkf	Natural logarithm of the gross formulation of fixed capital in Mexico, at 2008 prices.	INEGI
lnsalaries	Natural logarithm of the average daily salary by research institution, as registered in the Mexican Social Security Institute (IMSS). This series was deflated with the INPC base 2008 = 100.	National Minimum Wage Commission
SNIPEA	Members of the SNI per thousand members of the overall Economically Active Population in Mexico. This indicator is designed to measure the relative weight of human capital dedicated to existing R&D activities in Mexico, in comparison to the general population that develops some kind of economic activity or that has the potential to do so. This relationship is illustrated in the following expression: SNIPEA = SNI/PEA×10000	The source for SNI is CONACYT and for PEA is INEGI.
invpat	Inventive activity for patents. Measures patent applications by Mexican nationals by each 10,000 residents. The information sourced from the IMPI corresponds to the Mexican state of residency of the inventors named on applications for patent.	Mexican Industrial Property Institute (IMPI)
invmod	Inventive activity for utility models. Measures utility model applications by Mexican nationals by each 10,000 residents. The information sourced from the IMPI corresponds to the Mexican state of residency of the inventors named on applications for utility models.	IMPI
invdes	Inventive activity for industrial designs. Measures industrial design applications by Mexican nationals by each 10,000 residents. The information sourced from the IMPI corresponds to the Mexican state of residency of the inventors named on applications for industrial designs.	IMPI
searches	Technological information searches undertaken by IMPI.	IMPI
exterpat	Technological indicator that measures the externalities for patents that a given Mexican research institution absorbs from those that originate in other research institutions. Constructed based on the sum of the patents owned by all Mexican research institutions without considering the Mexican state under evaluation.	Authors’ elaboration based on IMPI data
extermod	Technological indicator that measures the externalities for utility models that a given Mexican research institution absorbs from those that originate in other research institutions. Constructed based on the sum of the patents owned by all Mexican research institutions without considering the Mexican state under evaluation.	Authors’ elaboration based on IMPI data
exterdis	Technological indicator that measures the externalities for industrial designs that a given Mexican research institution absorbs from those that originate in other research institutions. Constructed based on the sum of the patents owned by all Mexican research institutions without considering the Mexican state under evaluation.	Authors’ elaboration based on IMPI data
lnspend	Indicator that measures the contribution of technological efforts undertaken by Mexican research institutions located in a given state. Constructed based on the sum of R&D spending evaluated for the entire stock of research institutions without considering the Mexican state under evaluation.	Authors’ elaboration based on CONACYT data
indexpat	Index of patent concentration, defined as the relevance of patents in relation to the total of all forms of intellectual property.	Authors’ elaboration based on IMPI data
indexmod	Index of utility model concentration, defined as the relevance of patents in relation to the total of all forms of intellectual property.	Authors’ elaboration based on IMPI data
indexdes	Index of industrial design concentration, defined as the relevance of patents in relation to the total of all forms of intellectual property.	Authors’ elaboration based on IMPI data
hhp	Hirschman-Herfindahl index modified for patents.	Authors’ elaboration based on IMPI data
hhm	Hirschman-Herfindahl index modified for utility models.	Authors’ elaboration based on IMPI data
hhd	Hirschman-Herfindahl index modified for industrial designs.	Authors’ elaboration based on IMPI data

Source: Authors’ elaboration.

For the relationship of inventiveness indices (models 1–3):

$${lngdp}_{it}=a_{it}+\alpha {lnfbkf}_{it}+\beta {lnsalaries}_{it}+{\gamma invpat}_{it}\times {SNIPEA}_{it}+{\delta lnspend}_{it}+u_{it}$$

$${lngdp}_{it}=a_{it}+\alpha {lnfbkf}_{it}+\beta {lnsalaries}_{it}+{\gamma invmod}_{it}\times {SNIPEA}_{it}+{\delta lnspend}_{it}+u_{it}$$

$${lngdp}_{it}=a_{it}+\alpha {lnfbkf}_{it}+\beta {lnsalaries}_{it}+{\gamma invdis}_{it}\times {SNIPEA}_{it}+{\delta lnspend}_{it}+u_{it}$$

For the relationship of IP externalities (models 1–6):

$${lngdp}_{it}=a_{it}+\alpha {lnfbkf}_{it}+\beta {lnsalaries}_{it}+{\gamma exterpat}_{it}+u_{it}$$

$${lngdp}_{it}=a_{it}+\alpha {lnfbkf}_{it}+\beta {lnsalaries}_{it}+{\gamma extermod}_{it}+u_{it}$$

$${lngdp}_{it}=a_{it}+\alpha {lnfbkf}_{it}+\beta {lnsalaries}_{it}+{\gamma exterdes}_{it}+u_{it}$$

For the relationship of technological search information (7–8):

$${lngdp}_{it}=a_{it}+\alpha {lnfbkf}_{it}+\beta {lnsalaries}_{it}+{\gamma searches}_{it}+{\delta lnspend}_{it}+u_{it}$$

For the relationship of concentration indices modified by IP (models 9–11):

$${lngdp}_{it}=a_{it}+\alpha {lnfbkf}_{it}+\beta {lnsalaries}_{it}+{\gamma indexpat}_{it}+u_{it}$$

$${lngdp}_{it}=a_{it}+\alpha {lnfbkf}_{it}+\beta {lnsalaries}_{it}+{\gamma indexmod}_{it}+u_{it}$$

$${lngdp}_{it}=a_{it}+\alpha {lnfbkf}_{it}+\beta {lnsalaries}_{it}+{\gamma indexdes}_{it}+u_{it}$$

For the relationship of the Hirschman-Herfindahl indices modified by IP (models 12–14):

$${lngdp}_{it}=a_{it}+\alpha {lnfbkf}_{it}+\beta {lnsalaries}_{it}+{\gamma hhp}_{it}+u_{it}$$

$${lngdp}_{it}=a_{it}+\alpha {lnfbkf}_{it}+\beta {lnsalaries}_{it}+{\gamma hhm}_{it}+u_{it}$$

$${lngdp}_{it}=a_{it}+\alpha {lnfbkf}_{it}+\beta {lnsalaries}_{it}+{\gamma hhd}_{it}+u_{it}$$

Where the first term (*u*_*it*_ = *μ*_*i*_+*v*_*it*_) is the non-observable effect that captures the heterogeneity between the Mexican states included in the study, which can be treated as fixed or random. The second term is the residual stochastic component that includes unexplained spatial and temporal variance, with the assumption that it is independent and identically distributed with a mean of zero and constant variance. The subindexes *i* and *t* identify Mexican states and time, respectively. The explanatory variables and the explained variable are described in Table 1. The interaction of the variables *invpat*×*SNIPEA*, *invmod*×*SNIPEA*, y *invdis*×*SNIPEA* is adapted for the present research and supported by studies conducted by Rajan and Zingales [66], Soukiazis and Antunes [67], and Hu and Png [68], which estimate economic growth production functions. In Rajan and Zingales [66], the external dependency of an industry and the economic development of a country were interrelated; in Soukiazis and Antunes [67], an interaction was observed between the variables of human capital and international trade; and Hu and Png [68] established an interconnection between patent-intensive industries and industries with effective patent rights.

The inclusion of this set of variables in the present study is based on the premise that the Mexican states with a larger number of SNI members have greater incentives to generate inventive activity and therefore these states tend to generate higher economic growth rates in comparison to others. This argument is based on the fact that the SNI conceives of its researchers’ inventive activity to be a measurable product, given that the SNI considers inventive activity in making decisions about who to admit, maintain, and promote as members, and to whom to award monthly stimulus payments.

The indices of concentration employed in the model are based on those which have been constructed in prior analyses of the Mexican economy. These indicators are comprehensively described by Carranco and Godínez [69], which is essentially an adaptation of the work of Crocco *et al*. [70]. The indices developed in these prior studies and utilized in the present analysis employ modified Gini concentration indicators with a bias correction to measure the degree of skilled employment in diverse economic sectors in the area surrounding Azcapotzalco in Mexico City, Mexico. Due to the appropriateness of these indicators’ composition, it was decided to extrapolate them for use in the present case study of IP utilization by SNI researchers in Mexico.

The indicators in their abstract form are coefficients of relative national participation (RNP), which measures the contribution of variable (E) at the local level (j), as well as with the relationship of variable (E) with the larger region (R). The variable (E) has a subclassification (i).


$$PRN=\frac{E_{j}^{i}}{E_{R}^{i}};0\leq PRN\leq 1$$ (10)

The coefficients were adapted for the present study as follows: E together with the different types of IP analyzed, (i) utilization of each form of IP (patent, utility model, industrial design), by a given Mexican research institution (J) and by country (R), which in this case is Mexico. The location coefficient of the area of knowledge (QLR) displays the specificity of a sector in a determined geographical area. This coefficient indicates that the activity is of low relevance for the subset and for the set when it is positive but less than the unit. If the coefficient is superior to the unit this indicates greater relevance for the set.


QLR=EjiEjERiER;0<QLR (11)

In the specification adapted for the present analysis, the relevance of each form of IP is measured by Mexican state. Furthermore, each form of IP is weighed to determine its importance relative to the other IP forms. Finally, the modified Hirschman-Herfindahl is used as a coefficient to demonstrate the weight of these classifications in the local territorial structure, correcting for relative participation with participation in the set by the values employed for participation in the subset. The value of this coefficient should be superior or close to the average corrected by the first standard deviation, and it should also be positive.


$$HH=\frac{E_{j}^{i}}{E_{R}^{i}}-\frac{E_{j}}{E_{R}};HH\in R$$ (12)

As adopted, this indicator demonstrates the relevance of each form of IP protection. The first term shows the weight of each type of IP for Mexican research institutions, while the second coefficient displays the weight of the entire structure of IP for these institutions. We developed these indicators to measure the contribution made by each type of IP to GDP growth in Mexico, as well as to detect if the incentives associated with the utilization of the different forms of IP are appropriate.

We decided to focus on patents, utility models and industrial designs in relation to GDP growth because these three forms of IP are designed to protect innovative ideas. This stands in contrast to other IP mechanisms such as trademarks or copyright, which generally are not as closely associated with innovations in processes, products, or services. One of the advantages of patents is that in addition to being an indicator of inventive activity, patent documents and statistics are available for consultation by any economic agent. Additionally, patents allow for the discernment of researcher competence in different economic areas, because patent documents contain information about named inventors and demonstrate their scientific and technical expertise [71].

Despite the established links between patents, utility models and industrial designs and innovation, it is important to note that sometimes obtaining IP protection does not result in effective commercialization. In their work, Webster and Jensen [72] highlight the limitations of public research centers, individual inventors and small and medium-sized companies in products manufacturing and marketing, which together cause patents to have a low predictive effect on commercialization strategies. Meanwhile, studies by Aristizábal-Mesa *et al*. [73] and García [27] demonstrate that patents frequently operate to limit the entrance of new companies into a given industry, due to the costs involved in conducting R&D to invent around patent rights or obtain licenses to use inventions owned by established companies. Furthermore, patents may function to reduce the number of competitors in a given economic sector where there is monopolistic concentration of IP rights owned by a small number of firms.

It is also important to recognize that different resources are needed to obtain patents in comparison to utility models and industrial designs. The investment of time required to obtain a patent in Mexico is significant, requiring between two and five years, during which a rigorous examination is conducted by IMPI. In addition, the administrative costs for patents are higher than for utility models or industrial designs. These latter forms of IP can also be obtained in a shorter amount of time. Finally, it is notable that each of the three forms of IP examined in the present study covers a different set of products and services.

In the Mexican context, since the end of the first decade of the 2000s until 2018, CONACYT supported the commercialization of academic research by creating Technology Transfer Offices (TTOs), with the aim for these to operate as interlocutors between academia and the private sector in matters of technology transfer. Subsequently, CONACYT has begun to focus its efforts on developing a system of science and technology based on the quintuple helix model. The database utilized for the present analysis corresponds to a panel data structure that covers the period from 2003–2012, with a historical series of 10 annual data points and 32 transversal units represented by Mexican research institutions, for a total of 320 observations.

For the establishment of an appropriate econometric model, we conducted tests to guide the specification process, focusing on potential analytical options including OLS, random effects, or fixed effects. Nevertheless, due to the use of panel data, it remained possible that certain problems could arise, such as contemporaneous correlation, serial correlation, unit roots, and heteroskedasticity. The first three of these problems can be minimized in panel data by focusing on a short period of time. Torres [74] suggests that contemporaneous correlation can have a serious impact on macropanel inferences for periods between 20 and 30 years, but that employing structured micropanels with reduced intervals can reduce these detrimental effects. However, it was not possible to examine and address correlational problems with precision, due to the fact that the transverse units are superior to the length of time, or because both are of a relatively small size [75–77]. In order to correct for heteroscedasticity, two primary options exist. First, it is possible to use the heteroskedasticity-consistent covariance matrix estimator proposed by White [78], which consists of generating robust standard errors that are generally larger than those resulting from the ordinary least squares method. However, while this strategy produces consistent estimators when conditional heteroskedasticity is unknown, it is not efficient since it does not encompass the property of minimum variance.

The second approach to correct for heteroskedasticity involves the use of the feasible generalized least squares (FGLS) and the panel-corrected standard error (PCSE) procedures. These methods typically produce consistent estimators with minimum variance. However, there exists an ongoing debate about the precision of the FGLS and PCSE methods, in which the strengths and weaknesses of each relative to the other is contested [77, 79–83]. In order to avoid this debate, we utilized both the FGLS and PCSE approaches to correct for heteroskedasticity.

## Results and discussion

The present analysis focused on measuring the magnitude of the impact of the relationship between different forms of IP obtained by SNI researchers and economic growth in Mexico. For patents, utility models, and industrial designs alike, we found a strong correlation between the number of applications filed and membership of Mexican research institutions registered by CONACYT (Table 2). The descriptive statistics for the variables employed in the regressions are displayed in Table 3.

**Table 2:** Matrix of correlations of the variables.

Variables	gdp	fbkf	salaries	invpat	invmod	invdes	spend	SNIPEA	searches	indexpat	indexmod	indexdes	hhp	hhm	hhd
gdp	1														
fbkf	0.5885	1													
salaries	0.6937	0.2308	1												
invpat	0.8723	0.5061	0.5526	1											
invmod	0.8908	0.5715	0.5397	0.9338	1										
invdes	0.8312	0.5043	0.4629	0.8989	0.8830	1									
spend	0.6588	0.2599	0.4272	0.7478	0.6494	0.6840	1								
SNIPEA	0.8357	0.4270	0.5174	0.9030	0.8677	0.8188	0.7412	1							
searches	0.8396	0.4061	0.5027	0.8878	0.8779	0.828	0.6983	0.9736	1						
indexpat	0.0282	-0.0169	0.1008	-0.027	-0.1285	-0.2026	0.0301	-0.0418	-0.0747	1					
indexmod	-0.1160	-0.0427	-0.1764	-0.155	-0.0615	-0.2004	-0.2456	-0.1093	-0.1092	-0.2547	1				
indexdes	0.1494	0.2292	-0.0264	0.2153	0.2251	0.4099	0.3414	0.1707	0.1777	-0.509	-0.4408	1			
hhp	0.0235	-0.0507	0.1625	0.1408	0.0206	-0.2776	0.0665	0.1311	0.0823	0.4485	0.0636	-0.4402	1		
hhm	0.8097	0.4780	0.5022	0.7888	0.9179	0.7159	0.4745	0.7796	0.8021	-0.1078	0.0263	0.1220	0.0859	1	
hhd	0.8031	0.4498	0.4398	0.8339	0.8292	0.9755	0.6886	0.7837	0.8015	-0.2294	-0.2184	0.4404	-0.3653	0.6818	1

Source: Authors’ elaboration.

**Table 3:** Descriptive statistics of the variables.

Variable	Mean	Standard Deviation	Minimum	Maximum
lngdp	12.4377	0.8169	10.9685	14.6079
lnfbkf	8.0062	0.8608	4.9273	10.3530
lnsalaries	11.1881	0.1631	10.8535	11.9858
invpat	0.0024	0.0049	0.0000	0.0426
invmod	0.0013	0.0024	0.0000	0.0162
invdes	0.0040	0.0086	0.0000	0.0610
SNIPEA	2.6316	2.8324	0.1429	15.8660
searches	57.2656	185.6009	0.0000	1309
lnexterpat	5.3082	1.0507	1.6094	6.8320
lnextermod	4.7340	1.0088	1.0986	5.8464
lnexterdes	5.6101	1.4274	1.3863	7.3238
lnspend	21.0446	1.5339	1.6094	23.0426
indexpat	1.2083	0.7326	0.0000	3.4850
indexmod	4.4664	3.7951	0.0000	22.0980
indexdes	2.0650	1.5089	0.0000	7.0655
hhp	0.0000	0.0157	-0.0882	0.0562
hhm	0.0692	0.1351	-0.0191	1.1231
hhd	0.0614	0.1382	-0.0126	0.8349

Source: Authors’ elaboration.

An F-test was conducted to select between fixed effects and OLS, as was an LM test to select between random effects and least-squares. Finally, a Hausman test was performed to decide between fixed and random effects. Results demonstrated that the fixed effects model was the appropriate specification. To detect the problem of heteroskedasticity, the Wald heteroskedasticity test was conducted for fixed effects, which was statistically significant at 1% (Tables 8 and 9 of S1 Appendix). Heteroskedasticity was subsequently addressed by employing the FGLS and PCSE modelling and estimation procedures.

Models 1 and 3 of Tables 4 and 5 demonstrate that the Mexican states that have the highest inventiveness activity, and which contain the greatest number of SNI members, on average achieve higher rates of economic growth. The exception to the overall findings is shown in model 2; utility models were not statistically significant at conventional levels using the FGLS technique. It is important to note that in a previous study of the patenting activity of SNI members, Millán-Quintero and Meza-Rodríguez [84] found that some Mexican states with a large percentage of patents do not have a large number of researchers who are members of the SNI. The states of Mexico City, Morelos, Nuevo León, Coahuila, Querétaro, Jalisco, and Chihuahua are the jurisdictions that contribute 90.73% of the generation of patents and these states also have a large number of SNI members, with the exception of Chihuahua and Coahuila. Furthermore, 59.19% of patents are assigned to institutions located in Mexico City, and the largest number of SNI members are based there.

**Table 4:** Fixed effects estimates with FGLS heterskedasticity correction.

Variable	Model 1	Model 2	Model 3	Model 4	Model 5	Model 6	Model 7	Model 8
	Coefficient	Coefficient	Coefficient	Coefficient	Coefficient	Coefficient	Coefficient (2003–2012)	Coefficient (2003–2008)
lnfbkf	0.0996*** (0.0055)	0.0997*** (0.0055)	0.0984*** (.0054)	0.0530*** (0.0059)	0.0937*** (0.0065)	0.0578*** (0.0062)	0.1011*** (0.0056)	0.0918*** (0.0057)
lnsalaries	0.3620*** (0.0973)	0.3828*** (0.0968)	0.3581*** (0.0971)	0.2242*** (0.0874)	0.3757*** (0.1013)	0.3775*** (0.0807)	0.3864*** (0.0968)	0.5494*** (0.0737)
invpat*SNIPEA	0.2404*** (0.0776)							
invmod*SNIPEA		0.5301 (0.3283)						
invdes*SNIPEA			0.1810*** (0.0375)					
lnspend	0.0232*** (0.0034)	0.0236*** (0.0033)	0.0238*** (0.0033)				0.0241*** (0.0034)	0.0075*** (0.0020)
exterpat				0.1376*** (0.0087)				
extermod					0.0695*** (0.0108)			
exterdes						0.1002*** (0.0077)		
searches							0.0000007 (0.0001)	0.0005*** (0.0002)
Constant	6.4051*** (1.0733)	6.1636*** (1.0675)	6.4460*** (1.0713)	8.0106*** (0.9667)	6.4333*** (1.1203)	6.4108*** (0.8887)	6.1020*** (1.0668)	4.6748*** (0.8224)
Test								
Wald	111971.06 [0.000]	99759.59 [0.000]	146226.37 [0.000]	136027.25 [0.000]	82122.05 [0.000]	117302.15 [0.000]	98041.03 [0.000]	109351.84 [0.000]
Observations	320	320	320	320	320	320	320	192

Source: Authors’ elaboration.

The probability variables are presented in brackets and the standard errors in parenthesis.

*10% Significance

**5% Significance

***1% Significance.

**Table 5:** Fixed effects estimates with PCSE heteroskedasticity correction.

Variable	Model 1	Model 2	Model 3	Model 4	Model 5	Model 6	Model 7	Model 8
	Coefficient	Coefficient	Coefficient	Coefficient	Coefficient	Coefficient	Coefficient (2003–2012)	Coefficient (2003–2008)
lnfbkf	0.0961*** (0.0079)	0.0963*** (0.0080)	0.0958*** (0.0079)	0.0561*** (0.0090)	0.0834*** (0.0088)	0.0626*** (0.0088)	0.0983*** (0.0081)	0.1031*** (0.0091)
lnsalaries	0.2969* (0.1764)	0.3069* (0.1756)	0.2980* (0.1761)	0.3677** (0.1840)	0.2889* (0.1754)	0.3654** (0.1702)	0.3155* (0.1757)	0.4232*** (0.1020)
invpat*SNIPEA	0.3669*** (0.0828)							
invmod*SNIPEA		0.8077** (0.3464)						
invdes*SNIPEA			0.2583*** (0.0430)					
lnspend	0.0185*** (0.0041)	0.0188*** (0.0041)	0.0188*** (0.0041)				0.0191*** (.0042)	0.0079*** (0.0021)
exterpat				0.1309*** (0.0134)				
extermod					0.0864*** (0.0151)			
exterdes						0.0936*** (0.0103)		
searches							-0.00003 (0.0001)	0.0004** (0.0002)
Constant	7.2591*** (1.9531)	7.1398*** (1.9436)	7.2429*** (1.9495)	6.4244*** (2.0335)	7.3920*** (1.9432)	6.5504*** (1.8863)	7.0210*** (1.9447)	5.9938*** (1.1428)
Test								
Wald	111680.4 [0.000]	99452.21 [0.000]	145942.06 [0.000]	135598.27 [0.000]	81895.36 [0.000]	116882.37 [0.000]	97719.87 [0.000]	109085.51 [0.000]
Observations	320	320	320	320	320	320	320	192

Source: Authors’ elaboration.

To explain the results, counterfactual policy experiments were conducted using the descriptive statistics of Table 2 and the coefficients from Tables 4 and 5. For example, if the average number of SNI members per economically active population in a given Mexican state was 2.63, an increase of the inventiveness activity captured in patents had a standard deviation of 0.0049 and for this same variable the regression coefficient was 0.2404. Multiplying the three figures by 100% expresses the result of an increase in income of 0.31% using the FGLS estimator and 0.47% using the PCSE estimator. In the case of the inventiveness activity for utility models, the increase was 0.51% using PCSE. For industrial designs, the increase using FGLS was 0.39% and 0.58% using PCSE. It was observed that for the period analysed, the average national annual economic growth rate in Mexico was 2.49%.

The findings suggest the existence of an interaction between innovation, activities undertaken by researchers dedicated to scientific and technological R&D, and GDP growth.

However, it is important to recognize that the volume of patent applications submitted to IMPI by Mexican nationals is low in comparison to figures from relatively wealthier countries. Between 2003 and 2012, patent applications filed in Mexico by Mexican nationals represented from 4–8% of total applications [30]. In the context of the present study, it is notable that SNI researchers face a dilemma when deciding between publishing scientific articles and filing patent applications because the latter is more expensive in terms of both time and money. Furthermore, the evaluation periods that the SNI follows are better aligned with the timeline associated with peer review and revision of scientific publications than with that of patent examination.

An analogous dilemma often leads Mexican inventors to protect their inventions through forms of IP alternative to patents. This is demonstrated by the fact that Mexican nationals were responsible for 80–92% of total utility model applications and 33–48% of total industrial design applications for the period of 2003 to 2012 [30]. Although the time required for patent examination is relatively long compared to that required by other forms of IP, if a granted patent is obtained and commercialization is achieved, the economic benefits can be significantly greater for both institutions and individual inventors in comparison to industrial designs and utility models.

The Mexican and international literature cited in this study reveals that there is a positive association between patenting activity and economic growth. Nevertheless, it is also important to recognize that the knowledge conveyed in academic journal articles represents basic science and can serve as a springboard for the development of innovations in their early stages. This has been the case when best practices were followed in certain countries with advanced scientific and technological sectors [85]. According to the empirical evidence from Mexico, there is a positive relationship between the publication of scientific articles and the inventive activity embodied in patents [26, 28, 86].

The R&D expenditure variable was positively associated with GDP growth rate, and its mean fell within the range found by prior analyses that focused on economic growth in Mexico. Interpretation of the data generated in the present study suggests that an increase of 1% in R&D expenditures leads to an increase of between 0.19% and 0.24% of GDP using the FGLS and PCSE estimators. The findings related to externalities revealed that the Mexican states that benefitted from activities undertaken outside of their borders–for instance when knowledge was shared through IP licensing or when goods and services were exchanged–also demonstrated a greater proclivity to file for IP protections. Furthermore, these Mexican states achieved higher rates of economic growth, especially in the area of patents, with a rate of 0.14%.

In contrast to these results, technological information searches were not found to be statistically significant at conventional levels for the period analysed. However, a regression analysis was conducted that covered the period between 2003 and 2008, in which the series demonstrated a positive tendency. This finding is consistent with the economic theory that postulates a positive link between externalities that support an innovation ecosystem and economic growth.

**Fig 1:**
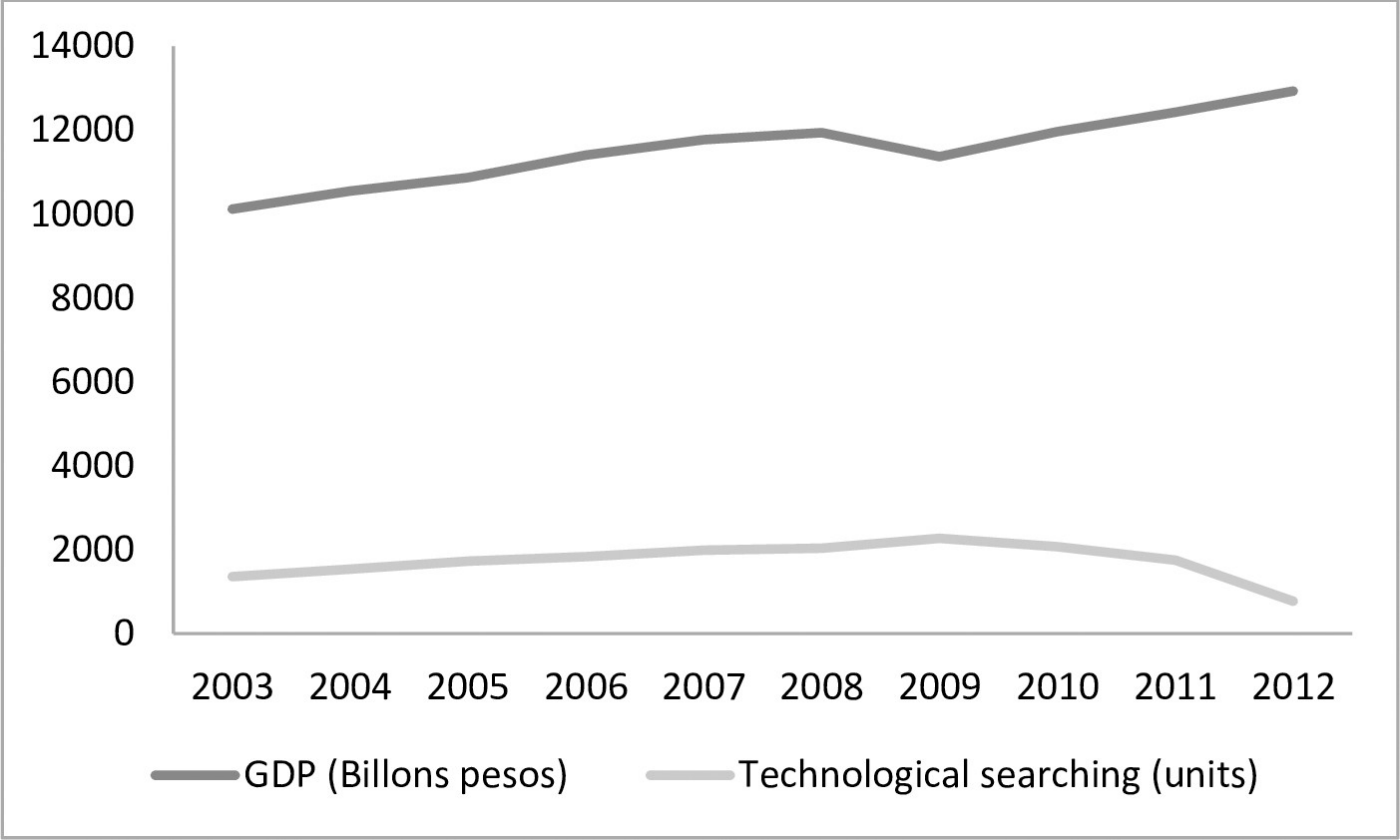
GDP and technological searches in Mexico. Source: Authors’ elaboration based on data from the National Institute of Statistics and Geography, Mexican Intellectual Property Institute.

As shown in Fig 1, from 2009 to 2012, there was a decrease in the demand for the technological information search services that IMPI provides. This may be explained by the fact that during this period some of IMPI’s services were replaced by those provided by public and private technology transfer offices, as well as by Patenting Centers established in CONACYT research institutions. For the present analysis, data were only available in relation to activities conducted by IMPI, and as such the activities of other actors in the Mexican innovation ecosystem were not studied. Therefore, the coefficient obtained could be underestimated, and it would be prudent to generate estimates with figures derived from the activities undertaken by other actors involved in this space.

With respect to IP concentration, the results demonstrated a positive association between the coefficient of patent concentration index and GDP, such that the Mexican states that most frequently obtain patents experienced the greatest positive impact in income. In contrast, the coefficient of the utility model concentration index was found to be negative. Specifically, while the patent and industrial design indicators demonstrated an upward pattern, the indicators for utility models reflected lower relative importance. Concentration in industrial designs resulted in the highest magnitude of impact on GDP, such that for an increase by one unit for this indicator, income increased by 0.87% (Tables 6 and 7). Overall, the findings from these concentration indexes revealed interesting patterns with respect to how participation in the IP system relates to rates of economic growth in Mexico.

**Table 6:** Fixed effects estimates with FGLS heterskedasticity correction.

Variable	Model 9	Model 10	Model 11	Model 12	Model 13	Model 14
	Coefficient	Coefficient	Coefficient	Coefficient	Coefficient	Coefficient
lnfbkf	0.1118*** (0.0059)	0.1075*** (0.0059)	0.1040*** (0.0061)	0.1134*** (0.0059)	0.1095*** (0.0059)	0.1073*** (0.0059)
lnsalaries	0.4765*** (0.1017)	0.3774*** (0.1034)	0.4768*** (0.1029)	0.4706*** (0.1010)	0.4873*** (0.1003)	0.5066*** (0.0995)
indexpat	0.0077* (0.0041)					
indexmod		-0.0023*** (0.0007)				
indexdes			0.0087*** (0.0022)			
hhpat				0.6414* (0.3436)		
hhmod					-0.1178*** (0.0451)	
hhdes						0.2028*** (0.0503)
Constant	5.5256*** (1.1251)	6.6790*** (1.1477)	5.5619*** (1.1396)	5.5886*** (1.1174)	5.4333*** (1.1104)	5.2254*** (1.1011)
Tests						
Wald	80944.08 [0.000]	83020.48 [0.000]	85445.15 [0.000]	75755.40 [0.000]	90944.06 [0.000]	103050.91 [0.000]
Observations	320	320	320	320	320	320

Source: Authors’ elaboration.

The probability variables are presented in brackets and the standard errors in parenthesis.

*10% Significance

**5% Significance

***1% Significance.

**Table 7:** Fixed effects estimates with PCSE heteroskedasticity correction.

Variable	Model 9	Model 10	Model 11	Model 12	Model 13	Model 14
	Coefficient	Coefficient	Coefficient	Coefficient	Coefficient	Coefficient
lnfbkf	0.1077*** (0.0080)	0.1043*** (0.0082)	0.0980*** (0.0085)	0.1080*** (0.0081)	0.1064*** (0.0081)	0.1046*** (0.0082)
lnsalaries	0.3595** (0.1737)	0.3022* (0.1725)	0.3197* (0.1751)	0.3260* (0.1733)	0.3237* (0.1725)	0.3250* (0.1723)
indexpat	0.0106 (0.0066)					
indexmod		-0.0034*** (0.0012)				
indexdes			0.0112 (0.0034)			
hhpat				0.6787* (0.3746)		
hhmod					-0.1159** (0.0566)	
hhdes						0.1756** (0.0689)
Constant	6.8599*** (1.9306)	7.5472*** (1.9191)	7.3515*** (1.9466)	7.2421*** (1.9256)	7.2815*** (1.9170)	7.2720*** (1.9152)
Tests						
Wald	80695.13 [0.000]	82755.24 [0.000]	85185.18 [0.000]	75497.32 [0.000]	90685.53 [0.000]	102778.18 [0.000]
Observations	320	320	320	320	320	320

Source: Authors’ elaboration.

The probability variables are presented in brackets and the standard errors in parenthesis.

*10% Significance

**5% Significance

***1% Significance.

Similar results were found by using the modified Hirschman-Herfindahl index. This type of indicator is another way to corroborate the pattern found among the Mexican states studied, where greater usage of certain forms of IP was associated with achieving higher rates of economic growth. Findings indicated that the Mexican states with the highest levels of patenting activity tended to exhibit the highest rates of economic growth. The same phenomenon occurred for industrial design protections, though the largest effect was seen for patents. In contrast, when applied to utility models the modified Hirschman-Herfindahl index expressed a negative link with GDP growth (Tables 6 and 7). This finding suggests that over time the importance of utility models has diminished in Mexico, and that the Mexican research institutions have shifted their focus towards obtaining other forms of IP protection.

## Conclusions

The question of how to design IP frameworks that would be appropriately suited to national needs has long been explored [87]. Several prior works have demonstrated that countries such as Japan initially focused on utility models as a means to promote endogenous innovation and technological development, and later shifted strategies to progressively encourage intensified patenting activity [41, 88]. In contrast, although Mexico has not yet achieved comparable levels of innovation as those observed in Japan during the latter country’s transition from utility models to patents, the results of the present study suggest that utility models appear to be losing importance in Mexico. Today, researchers employed in Mexican research institutions are increasingly opting for other forms of IP protection.

The findings of the present study demonstrate that at least as utilized by SNI researchers, patents and industrial designs can be understood as the forms of IP that most positively impact economic development in Mexico. This phenomenon was observed in the relationship between activities undertaken by SNI researchers, relevant externalities in the national innovation ecosystem, and indices of concentration and participation of IP rights. The results suggest that the mechanism employed by the SNI to evaluate its members should provide sufficient incentives for inventors to undertake the protection of their creations through patents and industrial designs.

An alternative assessment mechanism that the SNI could implement would ensure that the evaluation periods utilized to assess researcher productivity appropriately correspond to the relatively long duration of time required for patent prosecution. Doing so could encourage scientists working in different Mexican states to seek IP protection for their inventions in the form of patents, in addition to industrial design registrations. Overall, the results of this analysis demonstrated a positive relationship between usage of the IP system and economic growth. This information could encourage authorities in Mexico to increase R&D expenditure as a proportion of GDP, which on average is currently one of the lowest percentages among OECD member countries.

One of the limitations of the study was that it was not possible to determine in which sectors or in relation to which innovations the three forms of intellectual property are concentrated in such a way that the magnitude of the effects of economic growth can be measured. One way that future research could address this limitation would be to evaluate the effect of inventions protected under different IP regimes on economic activity, where the technologies in question have been developed in the context of the Nagoya Protocol. This is an increasingly important line of enquiry, given that the Protocol entered into force relatively recently, in 2014. The Nagoya Protocol is an international agreement whose purpose is to provide a framework under which countries aim to support the conservation of biodiversity in part by regulating access to and utilization of native genetic resources. Under the Nagoya Protocol model, firms may obtain significant economic benefits from the exploitation and commercialization of technologies in sectors including agriculture, health, nutrition, and cosmetics, where inventions that are based on native genetic resources are appropriately accessed and utilized.

A final limitation of the study was that only technological information searches realized by IMPI were considered, meaning that searches conducted by other actors such as consultants or TTOs were not included within the scope of analysis. The reason for this was that information on third party technological information searches was not available at the time of investigation. Therefore, it is possible that the effect of technological information searches on economic growth could be underestimated.

## Supporting information

S1 Appendix (DOCX)

S1 Data (XLSX)
