# Supplementary material for: Effects of the utilization of intellectual property by scientific researchers on economic growth in Mexico
Source: PLoS One. 2021 Oct 13;16(10):e0258131. doi: 10.1371/journal.pone.0258131 (PMC8513833; doi:10.1371/journal.pone.0258131)
Supplement: S1 Appendix — (DOCX) [file pone.0258131.s001.docx]

**S1 Appendix**

**Table 8. Tests for models 1-8.**

| **Estimation Method** | **Test** | **Model 1** | **Model 2** | **Model 3** | **Model 4** | **Model 5** | **Model 6** | **Model 7** | **Model 8** |
| --- | --- | --- | --- | --- | --- | --- | --- | --- | --- |
| OLS | F | 216.76 [0.000] | 218.28 [0.000] | 220.09 [0.000] | 317.85 [0.000] | 320.31 [0.000] | 314.34 [0.000] | 231.16 [0.000] | 158.49 [0.000] |
| Random Effects | Wald | 180.09 [0.000] | 179.53 [0.000] | 181.20 [0.000] | 242.34 [0.000] | 166.56 [0.000] | 211.46 [0.000] | 178.56 [0.000] | 180.14 [0.000] |
|  | LM | 497.84 [0.000] | 506.02 [0.000] | 497.14 [0.000] | 468.54 [0.000] | 472.60 [0.000] | 474.25 [0.000] | 515.75 [0.000] | 189.04 [0.000] |
|  | Hausman | 138.6088 [0.000] | 136.2763 [0.000] | 139.8522 [0.000] | 180.4383 [0.000] | 170.4175 [0.000] | 172.9963 [0.000] | 140.9234 [0.000] | 105.7176 [0.000] |
| Fixed Effects | F | 51.48 [0.000] | 50.31 [0.000] | 51.69 [0.000] | 118.13 [0.000] | 71.29 [0.000] | 98.52 [0.000] | 49.56 [0.000] | 50.82 [0.000] |
|  | Wald heteroskedasticity | 1474.67 [0.000] | 1431.96 [0.000] | 1713.77 [0.000] | 1005.70 [0.000] | 754.50 [0.000] | 2696.41 [0.000] | 1433.30 [0.000] | 2874.45 [0.000] |
|  | Fixed effects F | 309.9355 [0.000] | 305.2583 [0.000] | 306.9276 [0.000] | 371.6161 [0.000] | 291.4054 [0.000] | 341.0965 [0.000] | 290.3336 [0.000] | 328.4573 [0.000] |

Source: Authors’ elaboration.

**Table 9. Tests for models 9-14.**

| **Estimation Method** | **Test** | **Model 9** | **Model 10** | **Model 11** | **Model 12** | **Model 13** | **Model 14** |
| --- | --- | --- | --- | --- | --- | --- | --- |
| OLS | F | 258.16 [0.000] | 257.91 [0.000] | 259.41 [0.000] | 277.69 [0.000] | 290.71 [0.000] | 320.44 [0.000] |
| Random Effects | Wald | 154.72 [0.000] | 156.87 [0.000] | 157.30 [0.000] | 150.66 [0.000] | 151.29 [0.000] | 161.75 [0.000] |
|  | LM | 577.05 [0.000] | 584.98 [0.000] | 563.73 [0.000] | 471.73 [0.000] | 666.45 [0.000] | 600.31 [0.000] |
|  | Hausman | 113.6151 [0.000] | 114.7337 [0.000] | 124.7658 [0.000] | 150.2388 [0.000] | 110.4103 [0.000] | 118.7065 [0.000] |
| Fixed Effects | F | 54.08 [0.000] | 56.32 [0.000] | 57.13 [0.000] | 54.37 [0.000] | 54.10 [0.000] | 54.76 [0.000] |
|  | Wald heteroskedasticity | 1400.63 [0.000] | 2073.86 [0.000] | 1402.80 [0.000] | 1585.65 [0.000] | 1618.06 [0.000] | 1753.59 [0.000] |
|  | Fixed effects F | 307.5566 [0.000] | 312.5429 [0.000] | 312.9427 [0.000] | 292.0075 [0.000] | 281.5756 [0.000] | 262.4624 [0.000] |

Source: Authors’ elaboration
